# Central extracorporeal circulatory life support (cECLS) in selected patients with critical cardiogenic shock

**DOI:** 10.3389/fcvm.2023.1142953

**Published:** 2023-04-17

**Authors:** Leonie Schmack, Bastian Schmack, Maria Papathanasiou, Fadi Al-Rashid, Alexander Weymann, Nikolaus Pizanis, Markus Kamler, Arjang Ruhparwar, Tienush Rassaf, Peter Luedike

**Affiliations:** ^1^ Department of Cardiology and Vascular Medicine, West German Heart and Vascular Center, Medical Faculty, University Hospital Essen, Essen, Germany; ^2^ Department of Thoracic and Cardiovascular Surgery, West German Heart and Vascular Center Essen, University Hospital Essen, University Duisburg-Essen, Essen, Germany

**Keywords:** cardiogenic shock, ECLS, VA-ECMO, vascular access, surgical access

## Abstract

**Background:**

Percutaneous extracorporeal life support (pECLS) is increasingly applied in cardiogenic shock (CS) despite a lack of evidence from randomized trials. The in-hospital mortality rate of pECLS still reaches up to 60%, while vascular access site complications remain a shortcoming. Surgical approaches with central cannulation for ECLS (cELCS) have emerged as a bail-out option. To date, no systematic approach exists that allows a definition of inclusion or exclusion criteria for cECLS.

**Methods and results:**

This single-center, retrospective, case-control study includes all patients fulfilling criteria for CS at the West German Heart and Vascular Center Essen/Germany between 2015 and 2020 who underwent cECLS (*n* = 58), excluding post-cardiotomy patients. Seventeen patients received cECLS (29.3%) as a first-line treatment strategy and 41 patients as a second-line strategy (70.7%). The main complications leading to the use of cECLS as a second-line strategy were limb ischemia (32.8%) and ongoing insufficient hemodynamic support (27.6%). The first-line cECLS cohort showed a 30-day mortality rate of 53.3% that was constant during follow-up. The 30-day mortality rate of secondary cECLS candidates was 69.8% and the rate at 3 and 6 months was 79.1%. Younger patients (<55 years) were more likely to exhibit survival benefit with cECLS (*p* = 0.043).

**Conclusion:**

Surgical cECLS in CS is a feasible therapy for highly selected patients with hemodynamic instability, vascular complications, or peripheral access site limitations as complementary strategy in experienced centers.

## 1. Introduction

Cardiogenic shock (CS) represents the most severe form of acute heart failure syndrome and is associated with high mortality rates ranging from 30% to 60% (1). Minimally invasive peripheral placement of percutaneous ventricular assist devices (pVADs) or short-term veno-arterial extracorporeal membrane oxygenation (VA-ECMO), also referred to as peripheral extracorporeal life support (pECLS), by cannulation of the femoral artery and vein, are increasingly applied first-line approaches for establishing an immediate hemodynamic support in CS (2). Despite the increasing use of peripheral VA-ECMO/ECLS with limited data from randomized trials and advances in critical care management and technology, the in-hospital mortality rate of patients treated with pECLS still reaches up to 60% and has remained stable during the last decade (3–5).

Different pVADs are available. Historically, the intra-aortic balloon pump was a first-line pVAD in infarct-related CS, but it is no longer recommended for this indication on the basis of randomized data (2, 6). Microaxial flow pumps (e.g., Impella™) are commonly applied as a short-term therapy in SCAI stage C and D limited to reversible causes, in high-risk coronary interventions, or in transplant and durable VAD candidates (2, 7, 8). pECLS is used for SCAI stage C, D, and E with combined respiratory insufficiency, also limited to reversible causes or for transplant or durable VAD candidates, and might be established in select patients with refractory cardiac arrest as extracorporeal cardiopulmonary resuscitation (2, 7, 8). Numerous trials are ongoing or were recently completed to prove the superiority of mechanical circulatory support compared with the sole best medical treatment, e.g., ECLS-SHOCK (NCT03637205), DANGERshock (NCT0163350), ALLOASSIST (NCT03528291), and UNLOAD-ECMO (NCT05577195) (5).

Until evidence can guide therapy for pVADs or pECLS, an interdisciplinary shock team must be put in place, assessing the severity of CS and comorbidities to evaluate individual risks and benefits (2).

Percutaneous approaches for ECLS are often limited by vascular access complications or limb ischemia. The latter might be avoided by the application of a selective antegrade leg perfusion cannula or by cannulation of a surgical arterial graft. In addition, frequent complications in pECLS are severe pulmonary edema due to an increase in afterload leading to an incomplete left ventricular unloading and might be limited by insufficient overall cardiocirculatory support (3, 9). If an additional microaxial device for selective left ventricular unloading on top of pECLS is not sufficient (e.g., the ECMELLA concept, also referred to as ECPELLA) or technically not feasible, percutaneous therapeutic opportunities are fully exploited.

Surgical vascular access to provide central cannulation of the cardiovascular system has emerged as an optional escalation concept in this end-stage CS population in heart failure centers. Typically, cECLS is used in postcardiotomy shock with an already established thoracotomy. In contrast, neither evidence-based systematic approaches nor algorithms exist that allow for a definition of inclusion criteria for cECLS therapy in patients with critical CS and/or who have experienced a failure of minimally invasive pECLS strategies.

Here, we aim to identify patient characteristics and inclusion criteria for high-risk patients with CS who might benefit from this last-resort therapy.

## 2. Methods

### 2.1. Patients

This is a single-center, retrospective, case-control study, including all patients fulfilling the criteria of CS at the West German Heart and Vascular center Essen (WHGZ) between 2015 and 2020 and who were treated with central ECLS (cECLS). Postcardiotomy patients were excluded from the study. We investigated patient characteristics at cECLS implementation as well as the reasons for secondary implantation of a cECLS if other assist devices were already in place. We also studied clinical assessment tools such as available hemodynamics, use of intravenous vasopressors or inotropes, antibiotics, and clinical course on intensive care unit if documentation was available. Follow-up time points were 30 days, 3 months, and 6 months after cECLS implantation. The study was approved by the local Ethics Committee of the University Duisburg-Essen (vote number 22-10BO).

### 2.2. Surgical ECLS techniques

**Figure 1:**
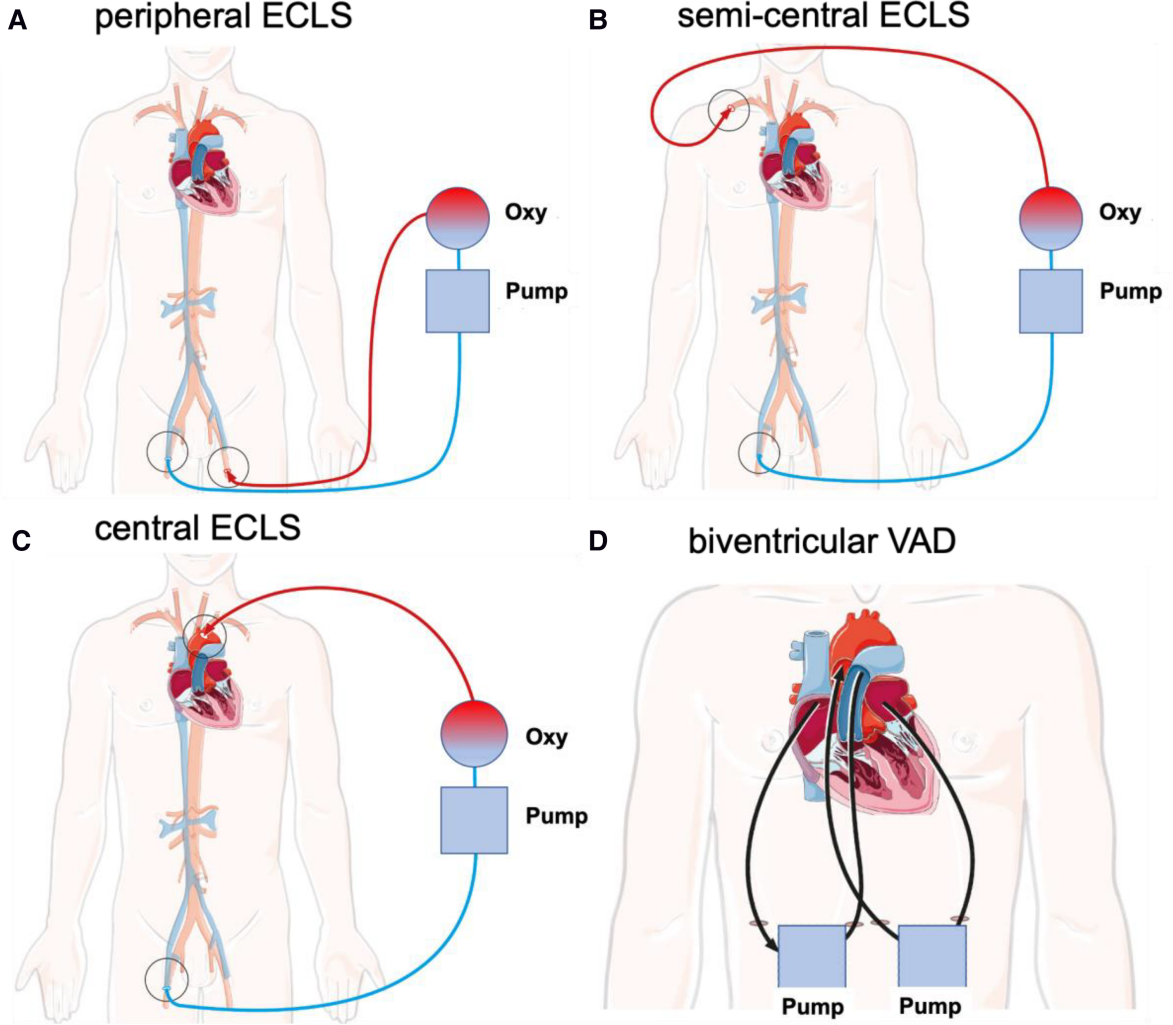
Schematic illustration of different cannulation techniques for interventional and surgical ECLS in adults as recommended by the ELSO. Deoxygenated blood (blue) is drained via the venous cannula under negative pressure. Blood is transferred through an oxygenator and oxygenated blood (red) is returned via the arterial cannula. (**A**) Peripheral ECLS. Insertion: Outflow via the femoral vein with inflow via the femoral artery (unilateral or bilateral). (**B**) Semicentral ECLS. Insertion: Outflow via the femoral vein or right atrium with inflow via the subclavian or axillary artery. (**C**) Central ECLS. Insertion: Outflow via the femoral vein or right atrium with inflow via the ascending aorta. (**D**) Biventricular VAD. Insertion: One outflow via the right atrium with inflow through the pulmonary trunk and a second outflow via the left atrium with inflow via the ascending aorta. cECLS, central venoarterial extracorporeal life support; ECLS, extracorporeal life support; pECLS, peripheral venoarterial extracorporeal life support; scECLS, semicentral venoarterial extracorporeal life support; BiVAD, temporary percutaneous biventricular assist device.

Possible interventional and surgical cannulation techniques for ECLS in adults as recommended by the Extracorporeal Life Support Organization (ELSO) are summarized in Table 1 and illustrated in Figure 1 (10).

**Table 1:** Different interventional and surgical techniques for ECLS in adults.

	pECLS	scECLS	cECLS	BiVAD
Cannulation	Peripheral	Semicentral	Central	Central
Configuration	Venoarterial			
Insertion (outflow → inflow)	Femoral vein → femoral artery	Femoral vein or right atrium → subclavian or axillary artery	Femoral vein or right atrium → ascending aorta	Right atrium → pulmonary trunk Left atrium → Ascending aorta
Cannula tips (outflow → inflow)	Inferior vena cava → abdominal aorta	Right atrium → ascending aorta	Right atrium → ascending aorta	Right atrium → pulmonary trunk Left atrium → Ascending aorta
Cannula size	Vein 18–29 Fr Arterial 15–22 Fr Reperfusion 8–9 Fr	Vein 18–29 Fr Right atrium 32–46 Fr Arterial 18–24 Fr	Right atrium 32–46 Fr Aortic 22–24 Fr	Left atrium 18–24 Fr Aortic 22–24 Fr
Type flow	Continuous centrifugal			
Flow	4–6 L/min (CI > 2.6 L/min/m^2^)			
Oxygenator	+	+	+	Optional
Support	Retrograde	Antegrade	Antegrade	Antegrade, selective
Advantages	• Emergency cannulation technique (e.g., extracorporeal cardiopulmonary resuscitation setting) • Possible cutdown	• No reperfusion cannula required	• Higher flow rates • Optimal drainage • Full body perfusion	• Blood flow via pulmonary vasculature • Cardiac unloading without extra strategy • Full body perfusion • Long-term bridging strategy
Disadvantages	• Cardiovascular support limited by peripheral arterial cannula size and venous drain capacity • Increase of cardiac afterload • Limb ischemia • Differential oxygenation between upper and lower body • No pulmonary circulation	• Not for emergency setting • Cardiovascular support limited by semicentral arterial cannula size and peripheral venous drain capacity • Surgery needed (local anesthesia) • No pulmonary circulation	• Emergency cannulation technique only in postcardiotomy setting • Surgery with sternotomy or resternotomy • No pulmonary circulation	• Not for emergency setting • Complex surgery
General complications	• Mechanical (e.g., pump failure, oxygenator failure, clots, cannula dislocations, etc.) • Hemorrhagic (e.g., bleeding, thrombocytopenia, etc.) • Neurologic (e.g., cerebral ischemia, infarction, etc.) • Renal (e.g., renal failure, continuous venovenous hemodialysis, etc.) • Cardiovascular (e.g., arrhythmia, tamponade, dissection, thromboembolism, etc.) • Pulmonary (e.g., pneumothorax, hemorrhage) • Infectious (e.g., sepsis) • Metabolic (e.g., hemolysis, hyperbilirubinemia, etc.)			
Specific complications	• Pulmonary congestion/edema • Central hypoxia with myocardial and/or cerebral ischemia • Lower limb complications (e.g., ischemia, compartment, etc.) • Left ventricular distension (esp. in case of aortic regurgitation) • Left ventricular thrombus • Harlequin syndrome	• Pulmonary congestion/edema • Upper limb complications (e.g., ischemia, compartment, etc.)	• Pulmonary congestion/edema • Sternum instability/infection • Wound healing disorder	• Left atrium thrombus • Sternum instability/infection • Wound healing disorder

cECLS, central venoarterial extracorporeal life support; CI, cardiogenic index; ECLS, extracorporeal life support; ECMELLA, ECLS and percutaneous microaxial pump; pECLS, peripheral venoarterial extracorporeal life support; BiVAD, temporary percutaneous selective biventricular assist device; scECLS, semicentral venoarterial extracorporeal life support.

In cECLS, blood drainage is performed by using a multistage venous cannula via the femoral vein introduced in the Seldinger technique. Alternatively, direct central cannulation of the right atrium is established. The advantages of cECLS are reliable venous drainage and arterial return with reduced impedance to the proximal aorta in antegrade fashion, allowing maximum flow rates without increasing the afterload. An alternative for left ventricular (LV) venting is cannulation of the right superior pulmonary vein. The main disadvantage of this is the invasive nature of cECLS, requiring sternotomy, potentially leading to subsequent complications such as bleeding, infection, resternotomy, aortic dissection, and thromboembolic events (11). As an alternative, semicentral ECLS (scECLS) with blood return in the ascending aorta via the axillary/subclavian artery is implemented. The advantage of scECLS is establishment without general anesthesia with only local anesthesia administered around the clavipectoral triangle. In select patients, a non-durable percutaneous biventricular assist device (BiVAD) is used as a bridge to decision or candidacy for left ventricular assist device (LVAD), heart transplant, or even recovery. Temporary BiVADs (e.g., Levitronix) provide circulatory support with an optional oxygenator in the right ventricular circuit (12). Until 2019, Levitronix had been applied as short-term circulatory support at our institution. For this reason, this subgroup was included in the cECLS cohort. BiVADs provide blood drainage from the right atrium to the pulmonary trunk as well as from the left atrium to the ascending aorta. The main advantages of BiVADs are complete cardiac unloading and maintaining the blood flow via pulmonary vasculature, avoiding the need for an oxygenator.

### 2.3. Statistics

Statistical analysis included sum, percentage, and mean for numerical variables, Pearson’s *χ*^2^ test for categorical data, independent *t*-test for quantitative data, and Kaplan–Meier estimator for mortality. The level of significance was *α* = 5%. Statistical analysis was performed using IBM SPSS Statistics 28 and Microsoft Excel version 16.59. In case of missing data, a listwise deletion of records was performed.

## 3. Results

### 3.1. Baseline characteristics of patients receiving central ECLS

Fifty-eight patients were retrospectively identified and included in the study. Basic patient characteristics at ECLS implantation are illustrated in Table 2. Primary condition leading to CS was acute coronary syndrome (*n* = 30, 51.7%), while non-acute coronary syndrome caused CS in 28 patients (48.3%). Half of the patients underwent successful resuscitation before implantation (50.0%, *n* = 29), and in 22.4% of patients (*n* = 13), the indication for pECLS was ongoing resuscitation, referred to as extracorporeal cardiopulmonary resuscitation (eCPR). The mean time of cardiopulmonary resuscitation before the establishment of eCPR was 40.3 min. Patients who underwent cECLS were grouped in SCAI stages C (first line *n* = 5; 29.4%/second line *n* = 15; 36.6%), D (first line *n* = 8; 47.1%/second line *n* = 19/46.3%), and E (first line *n* = 4; 23.5%/second line *n* = 7; 17.1%) without significant differences between groups.

**Table 2:** Baseline patient characteristics.

	Number	Mean (IQR)	Percent
Number	58		
Age (years)		54 (19)	
Male	44		76
**Cardiogenic shock**			
ST-elevation myocardial infarction acute coronary syndrome	25		43
Non-ST-elevation myocardial infarction acute coronary syndrome	5		9
Non-acute coronary syndrome CS	28		48
**Cardiopulmonary resuscitation**			
Extracorporeal cardiopulmonary resuscitation at insertion	13		22
Cardiopulmonary resuscitation before insertion	29		50
No cardiopulmonary resuscitation before insertion	16		28
Left ventricular ejection fraction	58		
HFpEF (≥50%)	0		0
HFmrEF (40%–49%)	3	42 (2.5)	
HFrEF (<40%)	55	17 (5.5)	
**Secondary diagnosis**			
Dilative cardiomyopathy	11		19
Ischemic cardiomyopathy	12		20
Myocarditis	6		10
Coronary artery disease	34		59
Peripheral artery disease	9		16
Hypertension	19		33
Arterial fibrillation	17		29
Diabetes mellitus	12		21
Obesity	11		19
Malignant disease	17		29

Data are presented as number, mean ± standard deviation (SD), and IQR, percent. ECLS, extracorporeal life support; pECLS, peripheral venoarterial extracorporeal life support.

### 3.2. Surgical ECLS strategy

Seventeen patients received cECLS (29.3%) as a first-line strategy, including four patients with surgical preparation and cannulation of the subclavian artery (scECLS 6.9%). The switch to cECLS as a second-line strategy due to pECLS failure was applied in 41 patients (70.7%). cECLS was intended as a bridge to decision in all patients. The mean time to switch to cECLS after pECLS insertion was 2 days (±67 h) (Table 3). The laboratory results of patients at surgical ECLS implantation are summarized in Table 4. Laboratory parameters were comparable between groups.

**Table 3:** ECLS implantation techniques.

ECLS implantation techinques	Number	Percent
Unifemoral pECLS	27	46
Bifemoral pECLS	14	24
Direct scECLS	5	9
Direct cECLS	12	21
**Surgical ECLS cannulation technique**		
cECLS	36	62
scECLS	6	10
BiVAD	16	28

Futile insertion of pECLS was rated as direct cECLS. cECLS, central venoarterial extracorporeal life support; ECLS, extracorporeal life support; BiVAD, temporary central selective biventricular assist device; pECLS, peripheral venoarterial extracorporeal life support; scECLS, semicentral venoarterial extracorporeal life support.

**Table 4:** Laboratory findings.

Laboratory results at surgical ECLS implantation	cECLS/scECLS implantation	Mean ± SD (IQR)	*p*-Value
Hemoglobin (mmol/L)		6.45 ± 1.43 (1.55)	
	First line	6.33 ± 1.22 (1.92)	0.66
	Second line	6.50 ± 1.52 (1.61)	
Partial thromboplastin time (sec)		59.5 ± 39.6 (32.0)	
	First line	56.2 ± 42.8 (13.8)	0.68
	Second line	61.0 ± 38.0 (32.2)	
Thrombocytes (10^9^/L)		145.6 ± 90.8 (146.0)	
	First line	159.8 ± 90.5 (148.0)	0.45
	Second line	139.7 ± 90.3 (152.0)	
White blood cells (10^9^/L)		14.9 ± 6.58 (7.7)	
	First line	14.1 ± 6.48 (7.44)	0.53
	Second line	15.3 ± 6.59 (7.34)	
Creatinine (µmol/L)		167.2 ± 64.2 (1.14)	
	First line	160.9 ± 62.8 (115.8)	0.50
	Second line	175.9 ± 65.4 (81.3)	
Glomerular filtration rate (ml/min/1.73 m^2^)		41.0 ± 14.2 (30.0)	
	First line	43.9 ± 14.1 (31.5)	0.45
	Second line	40.2 ± 14.1 (24.5)	
C-reactive protein (nmol/L)		1,026.7 ± 911.4 (1,166.7)	
	First line	1,028.6 ± 1,009.5 (1,219.0)	0.99
	Second line	1,028.6 ± 863.8 (1,071.4)	
Lactate (mmol/L)		5.60 ± 4.90 (5.43)	
	First line	6.80 ± 5.62 (6.00)	0.06
	Second line	4.30 ± 3.61 (3.65)	

Data are presented as mean ± standard deviation (SD) and IQR. ECLS, extracorporeal life support.

The main indication leading to cECLS was limb ischemia after femoral cannulation (although a selective limb perfusion cannula was inserted in 18 out of 19 patients) (*n* = 19, 32.8%). Hemodynamic instability led to cECLS switch in 16 patients (*n* = 27.6%). In four patients (6.9%), right ventricular failure was documented. Left ventricular congestion was the leading pathophysiology in three patients (5.2%). Acute bleeding was documented in seven patients (12.1%) and two patients presented an acute dislocation of one of the cannulas (3.4%). Some patients fulfilled more than one criterion for switch to cECLS. In five patients, a combination of Impella on top of ECLS (ECMELLA) was used (8.6%). In three patients with cECLS, surgical left ventricular venting was installed (5.2%). At the time of cECLS implantation, 27 (46.6%) patients fulfilled the criteria of multiorgan failure (first-line cECLS 58.8%, vs. second-line cECLS 41.5%, *p* = 0.25), with 58.6% of patients already receiving antibiotics (first-line cECLS 58.8% vs. second-line cECLS 58.5%, *p* = 0.80), and 29.3% undergoing continuous hemofiltration (first-line cECLS 17.6% CVVHD vs. second-line cECLS 34.1% CVVHD, *p* = 0.21). All patients in both cohorts required vasopressor and inotropic support at the time of cECLS implantation.

### 3.3. Outcome and follow-up

**Figure 2:**
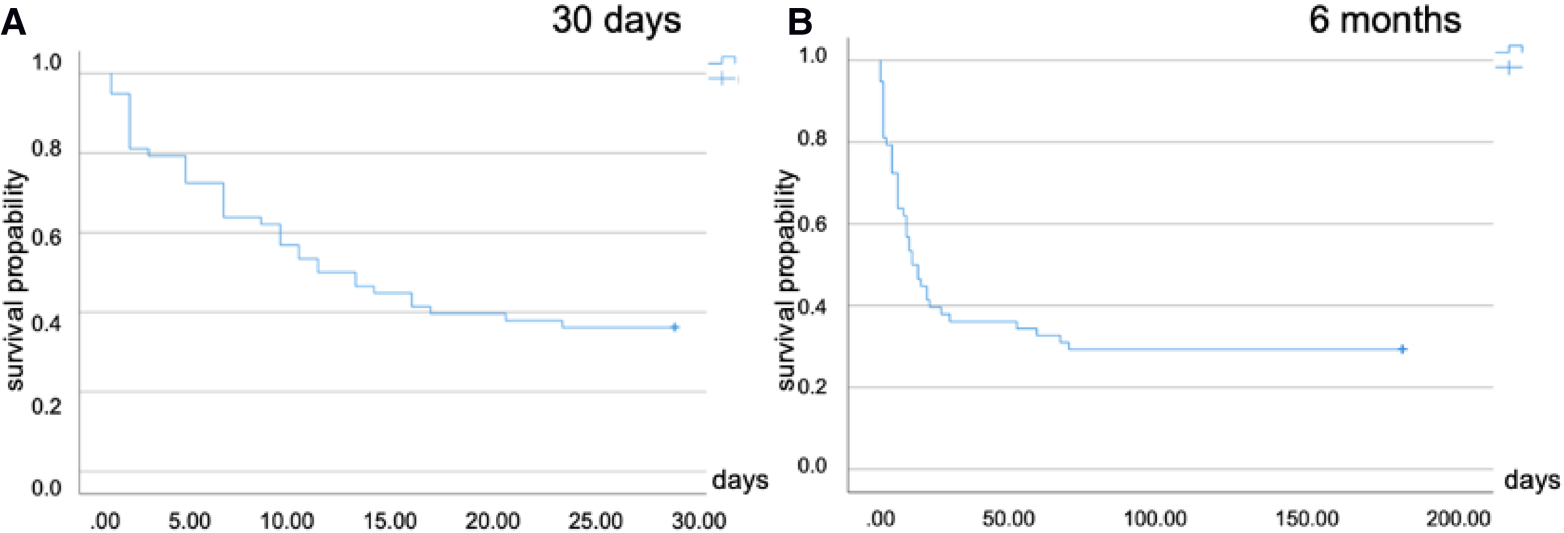
Kaplan–Meier plots for cumulative survival following surgical ECLS implantation. (**A**) Thirty-day mortality in the whole cohort (63.8%). (**B**) Six-month mortality (70.7%). ECLS, extracorporeal life support.

The age of non-survivors in the whole cohort was 56 ± 13 years. The main reason for death was multiorgan failure (53.4%, *n* = 31). Five patients died of intracranial bleeding (8.6%). One patient died because of ventricle rupture. The age of survivors was 49 ± 10 years. Following cECLS, 30-day mortality rate in the whole cohort was 63.8% (*n* = 37), corresponding to a survival rate of 36.2% (Figure 2A). The long-term mortality rate at 3 months was 70.7% (*n* = 41), without making any difference to the 6-month mortality rate (Figure 2B). Subdividing different cECLS implantation timing modalities, either secondary to pECLS failure or primary cECLS, the second-line strategy resulted in 30-day mortality rates of 69.8% and 79.1% at 3 and 6 months, respectively. First-line candidates exhibited a 30-day mortality rate of 53.3% without changes at 3- and 6-month follow-up interval. Younger patients (<median age of 55 years) were more likely to enjoy survival benefit after cECLS treatment (*p* = 0.043). In total, three first-line cECLS patients were bridged to LVAD (17.6%), and none of these patients died. In comparison, four second-line cECLS patients were treated with LVAD (9.7%), and one of these patients died 54 days after cECLS establishment. None of the patients in both cohorts received heart transplantation during the follow-up period.

## 4. Discussion

In this study, we identified patient characteristics and prerequisite criteria for patients who might benefit from surgical/central cannulation for ECLS. In reputed centers, cECLS appears to be a complementary strategy and a potential option for carefully selected patients in case they suffer from vascular complications, peripheral access site limitations, or hemodynamic instability.

According to the ELSO registry report, over 33,000 adults have been supported with ECLS for cardiac reasons since 1990, constantly increasing in frequency (13). The minimally invasive pECLS is regarded as a bail-out approach in refractory CS despite less evidence from randomized trials and the hemodynamic shortcomings of this therapy (5, 14–16). Surgical vascular access for ECLS with central cannulation of the cardiovascular system has emerged as an individual and almost experimental last-resort option, with even less evidence to be found in the literature (11). In an eCPR setting, the establishment of pECLS via ultrasound-guided stiff-wire peripheral cannulation is the preferred approach, with the exception of cardiac patients who had undergone sternotomy recently (thoracic cannulation recommended) (17). However, cECLS requires a cardiothoracic surgeon on site, new sternotomy, or at least surgical access to the axillary/subclavian artery. Because of this invasive nature, this strategy is poorly established in emergency situations (18).

While being a widely applied approach for hemodynamic support in CS, pECLS might not provide sufficient cardiovascular support, subsequently leading to pECLS-associated poor perfusion and typical harlequin syndrome (also referred to as North–South syndrome) (19). Complications such as thrombosis and thromboembolism as well as ischemia of the extremities are common. Accordingly, the main limitation of pECLS seems to be severe peripheral artery disease and subsequent limb ischemia despite selective limb perfusion cannulation (20).

The main reason for the switch to cECLS in our study was the presence of vascular complication. This finding gives rise to the hypothesis that upfront cECLS implantation might be a feasible first-line strategy in patients with known peripheral artery disease. Since a large number of pECLS devices are implanted in an emergency setting, a strategy such as primary cECLS needs to be clearly defined and investigated in a prospective fashion and is limited to only those sites where timely surgical support is available.

A hemodynamic aspect of pECLS treatment is that the increased afterload caused by the retrograde nature of the flow and small arterial cannulas might cause insufficient hemodynamic support, leading to pulmonary edema and prolonged weaning (21–24). Ongoing hemodynamic instability was one of the main reasons leading to a switch to cECLS in our study cohort. cECLS can help overcome some specific hemodynamic problems of pECLS, particularly a decrease of afterload, facilitating antegrade flow and additionally enabling optional left ventricular unloading. It might be hypothesized that an individual and differentiated hemodynamic approach with additional microaxial pumps, even if more invasive (e.g., ECMELLA), seems to be advantageous in mechanical ECLS for left ventricular unloading (25, 26). This concept is currently under investigation in the prospective, randomized UNLOAD-ECMO trial.

Major bleeding is reported to occur in approximately one-quarter of all ECLS candidates (27). In keeping with this trend, bleeding was a major issue in our pECLS cohort, which led to a central switch. Severe complications leading to cECLS escalation occurred in the acute phase after the implementation of pECLS. The statement that patients with known or acquired bleeding disorder might benefit from a primary/first-line cECLS strategy sounds hypothetical, but it might be a part of a decision algorithm.

The survival rate after 30 days in contemporary ECLS trials is reported to be as low as 50%, with an even reduced survival rate of only 23.5% at 1 year (5, 28). In our retrospective cohort, cECLS was associated with a 30-day mortality rate of 63.8%, rising to 70.7% after 6 months. The mortality rate among those patients who were treated with cECLS as a secondary strategy was higher than that among those treated with primary cECLS. In those who received cECLS as a first-line strategy, the long-term mortality rate was 53.3% after 6 months. In combination with the finding of younger patients (<55 years) to be more likely to enjoy survival benefit in our analysis, these data support the utility and feasibility of this concept in select patients.

The phenotype of CS can include right ventricular and/or left ventricular failure and subsequently necessitates an appropriate mechanical support. Right ventricular failure is characterized by central venous pressure (CVP) >15 mmHg, pulmonary pulsatility index <1.85, and right atrial to pulmonary capillary wedge pressure (PCWP) ratio (RA/PCWP) >0.8 (29). Left ventricular failure is defined as systolic blood pressure (SBP) below 90 mmHg or mean atrial pressure below 60 mmHg or more than 30 mmHg drop with inotropes/vasopressors, respectively. Additional parameters are cardiac index <2.2 L/min/m^2^ and cardiac power output <0.6 W and PCWP > 15 mmHg. A consideration of these invasive hemodynamic parameters can help identify patients with biventricular failure, although prospective data supporting this approach are not available (30).

### 4.1. Clinical flow chart

**Figure 3:**
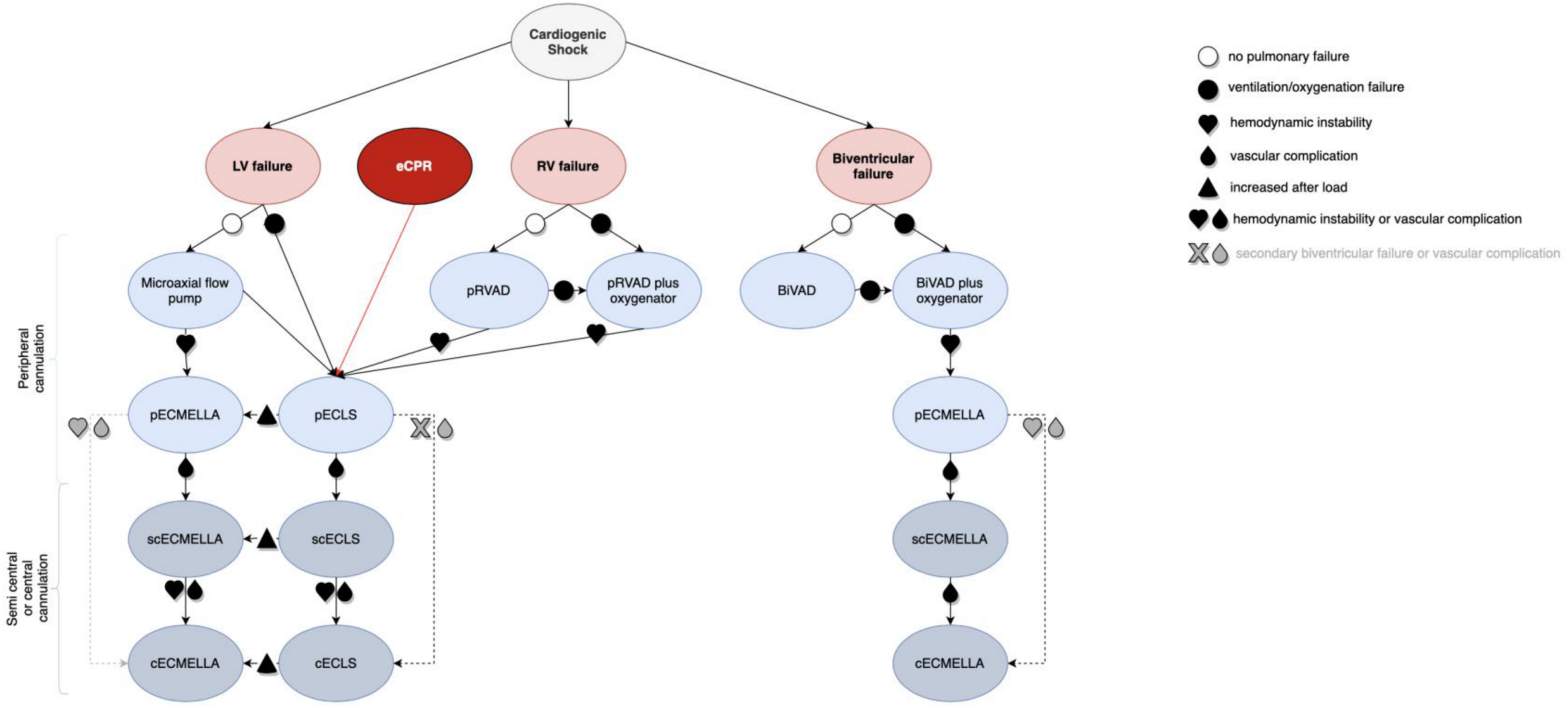
Decision algorithm for cannulation strategies in critical cardiogenic shock with the need for ECLS (visual take-home graphic). cECLS, central venoarterial extracorporeal life support; cECMELLA, central ECLS plus percutaneous microaxial pump; ECLS, extracorporeal life support; BiVAD, temporary percutaneous selective biventricular assist device; pECLS, peripheral venoarterial extracorporeal life support; pECMELLA, peripheral ECLS plus percutaneous microaxial pump; pRVAD, temporary percutaneous right ventricular support; scECLS, semicentral venoarterial extracorporeal life support; scECMELLA, semicentral ECLS plus percutaneous microaxial pump.

A flow chart to support individualized clinical decision-making for escalation strategies in CS is given in Figure 3. The basic consideration of the here proposed algorithm is that all ECLS therapies should be indicated as a bridge to heart and/or lung transplantation, durable mechanical circulatory support, recovery, or shared decision-making (31) to avoid therapeutic futility.

### 4.2. Limitations

The main limitation of this study is the retrospective, single-center design and the lack of digital patient charts during the analysis period. The mean age of the overall cohort was comparatively young, which represents a possible selection bias for this escalation therapy. Moreover, not all patients underwent comprehensive hemodynamic monitoring; therefore, decision-making in the current cohort could not be supplemented by additional data. Prospective randomized trials are needed to identify indications and cannulation strategies for cECLS therapy.

## 5. Conclusion

Surgical cannulation for central ECLS is a feasible therapeutic option for carefully selected patients presenting with refractory CS and might be implemented as an additional strategy in reputed centers.

## Data Availability

The original contributions presented in the study are included in the article/Supplementary Material, further inquiries can be directed to the corresponding author.

## References

[1] Chioncel O Parissis J Mebazaa A Thiele H Desch S Bauersachs J Epidemiology, pathophysiology and contemporary management of cardiogenic shock—a position statement from the Heart Failure Association of the European Society of Cardiology. Eur J Heart Fail. (2020) 22:1315–41. 10.1002/ejhf.1922 32469155

[2] Chieffo A Dudek D Hassager C Combes A Gramegna M Halvorsen S Joint EAPCI/ACVC expert consensus document on percutaneous ventricular assist devices. EuroIntervention. (2021) 17:e274–86. 10.4244/EIJY21M05_01 34057071 PMC9709772

[3] Becher PM Schrage B Sinning CR Schmack B Fluschnik N Schwarzl M Venoarterial extracorporeal membrane oxygenation for cardiopulmonary support. Circulation. (2018) 138:2298–300. 10.1161/CIRCULATIONAHA.118.036691 30571518

[4] Combes A Leprince P Luyt CE Bonnet N Trouillet JL Leger P Outcomes and long-term quality-of-life of patients supported by extracorporeal membrane oxygenation for refractory cardiogenic shock. Crit Care Med. (2008) 36:1404–11. 10.1097/CCM.0b013e31816f7cf7 18434909

[5] Ostadal P Rokyta R Karasek J Kruger A Vondrakova D Janotka M ECMO-CS Investigators. Extracorporeal membrane oxygenation in the therapy of cardiogenic shock: results of the ECMO-CS randomized clinical trial. Circulation. (2022). 147 (6):454–64. 10.1161/CIRCULATIONAHA.122.062949 36335478

[6] Thiele H Zeymer U Neumann FJ Ferenc M Olbrich HG Hausleiter J Intraaortic baloon support for myocardial infarction with cardiogenic shock. N Engl J Med. (2012) 367:1287–96. 10.1056/NEJMoa1208410 22920912

[7] Thiele H Jobs A Ouweneel DM Henriques JPS Seyfarth M Desch S Percutaneous short-term active mechanical support devices in cardiogenic shock: a systematic review and collaborative meta-analysis of randomized trials. Eur Heart J. (2017) 38:3523–31. 10.1093/eurheartj/ehx363 29020341

[8] Baran DA Grines CL Bailey S Burkhoff D Hall SA Henry TD SCAI clinical expert consensus statement on the classification of cardiogenic shock. Catheter Cardiovasc Interv. (2019) 94:29–37. 10.1002/ccd.28329. 31104355

[9] Lusebrink E Orban M Kupka D Scherer C Hagl C Zimmer S Prevention and treatment of pulmonary congestion in patients undergoing venoarterial extracorporeal membrane oxygenation for cardiogenic shock. Eur Heart J. (2020) 41:3753–61. 10.1093/eurheartj/ehaa547 33099278

[10] Gajkowski EF Herrera G Hatton L Velia Antonini M Vercaemst L Cooley E. ELSO Guidelines for adult and pediatric extracorporeal membrane oxygenation circuits. ASAIO J. (2022) 68(2):133–52. 10.1097/MAT.0000000000001630 35089258

[11] Saeed D Stosik H Islamovic M Albert A Kamiya H Maxhera B Femoro-femoral versus atrio-aortic extracorporeal membrane oxygenation: selecting the ideal cannulation technique. Artif Organs. (2014) 38:549–55. 10.1111/aor.12245 24392890

[12] Schlensak C Schibilsky D Siepe M Brehm K Klemm R von Wattenwyl R Biventricular cannulation is superior regarding hemodynamics and organ recovery in patients on biventricular assist device support. J Heart Lung Transplant. (2011) 30:1011–7. 10.1016/j.healun.2011.02.013 21489816

[13] Extracorporeal Life Support Organization. ECLS Registry Report, International Summary (2020). Available at: https://www.elso.org/Portals/0/Files/Reports/2021_October/International%20Report%20October_page1.pdf (Accessed July 17, 2022).

[14] Stretch R Sauer CM Yuh DD Bonde P. National trends in the utilization of short-term mechanical circulatory support: incidence, outcomes, and cost analysis. J Am Coll Cardiol. (2014) 64:1407–15. 10.1016/j.jacc.2014.07.958 25277608

[15] Cavarocchi NC. Introduction to extracorporeal membrane oxygenation. Crit Care Clin. (2017) 33:763–6. 10.1016/j.ccc.2017.06.001 28887925

[16] Rao P Khalpey Z Smith R Burkhoff D Kociol RD. Venoarterial extracorporeal membrane oxygenation for cardiogenic shock and cardiac arrest. Circ Heart Fail. (2018) 11:e004905. 10.1161/CIRCHEARTFAILURE.118.004905 30354364

[17] ELSO. ECPR supplement to the ELSO general guidelines (2013). Version 1.3. Available at: https://www. elso.org/Portals/0/IGD/Archive/FileManager/6713186745cusersshyerdocumentselsoguide linesforecprcases1.3.pdf.

[18] Biscotti M Bacchetta M. The “sport model”: extracorporeal membrane oxygenation using the subclavian artery. Ann Thorac Surg. (2014) 98:1487–9. 10.1016/j.athoracsur.2014.02.069 25282228

[19] Pasrija C Bedeir K Jeudy J Kon ZN. Harlequin syndrome during venoarterial extracorporeal membrane oxygenation. Radiol Cardiothorac Imaging. (2019) 1:e190031. 10.1148/ryct.2019190031 33778505 PMC7970096

[20] Avalli L Sangalli F Migliari M Maggioni E Gallieri S Segramora V Early vascular complications after percutaneous cannulation for extracorporeal membrane oxygenation for cardiac assist. Minerva Anestesiol. (2016) 82:36–43. PMID: 25907578

[21] Jayaraman AL Cormican D Shah P Ramakrishna H. Cannulation strategies in adult veno-arterial and veno-venous extracorporeal membrane oxygenation: techniques, limitations, and special considerations. Ann Card Anaesth. (2017) 20:S11–8. 10.4103/0971-9784.197791 28074818 PMC5299823

[22] Schmack B Seppelt P Weymann A Alt C Farag M Arif R Extracorporeal life support with left ventricular decompression-improved survival in severe cardiogenic shock: results from a retrospective study. PeerJ. (2017) 5:e3813. 10.7717/peerj.3813 28975053 PMC5624302

[23] Schrage B Becher PM Bernhardt A Bezerra H Blankenberg S Brunner S Left ventricular unloading is associated with lower mortality in patients with cardiogenic shock treated with venoarterial extracorporeal membrane oxygenation: results from an International, Multicenter Cohort Study. Circulation. (2020) 142:2095–106. 10.1161/CIRCULATIONAHA.120.048792 33032450 PMC7688081

[24] Weymann A Schmack B Sabashnikov A Bowles CT Raake P Arif R Central extracorporeal life support with left ventricular decompression for the treatment of refractory cardiogenic shock and lung failure. J Cardiothorac Surg. (2014) 9:60. 10.1186/1749-8090-9-60 24678718 PMC3974212

[25] Eulert-Grehn JJ Starck C Kempfert J Falk V Potapov E. ECMELLA 2.0: single arterial access technique for a staged approach in cardiogenic shock. Ann Thorac Surg. (2021) 11:e135–7. 10.1016/j.athoracsur.2020.06.084 32918864

[26] Ruhparwar A Zubarevich A Osswald A Raake PW Kreusser MM Grossekettler L ECPELLA 2.0-minimally invasive biventricular groin-free full mechanical circulatory support with Impella 5.0/5.5 pump and ProtekDuo cannula as a bridge-to-bridge concept: a first-in-man method description. J Card Surg. (2020) 35:195–9. 10.1111/jocs.14283 31609509

[27] Sy E Sklar MC Lequier L Fan E Kanji HD. Anticoagulation practices and the prevalence of major bleeding, thromboembolic events, and mortality in venoarterial extracorporeal membrane oxygenation: a systematic review and meta-analysis. J Crit Care. (2017) 39:87–96. 10.1016/j.jcrc.2017.02.014 28237895

[28] Chang CH Chen HC Caffrey JL Hsu J Lin JW Lai MS Survival analysis after extracorporeal membrane oxygenation in critically ill adults: a nationwide cohort study. Circulation. (2016) 133:2423–33. 10.1161/CIRCULATIONAHA.115.019143 27199466

[29] Korabathina R Heffernan KS Parachuri V Patel AR Mudd JO Prutkin JM The pulmonary artery pulsatility index identifies severe right ventricular dysfunction in acute inferior myocardial infarction. Catheter Cardiovasc Interv. (2012) 80:593–600. 10.1002/ccd.23309 21954053

[30] Kapur NK Thayer KL Zweck E. Cardiogenic shock in the setting of acute myocardial infarction. Methodist Debakey Cardiovasc J. (2020) 16:16–21. 10.14797/mdcj-16-1-16 32280413 PMC7137623

[31] Guglin M Zucker MJ Bazan VM Bozkurt B El Banayosy A Estep JD Venoarterial ECMO for adults: JACC scientific expert panel. J Am Coll Cardiol. (2019) 73:698–716. 10.1016/j.jacc.2018.11.038 30765037

